# Undertaking clinical audit, with reference to a Prescribing Observatory for Mental Health audit of lithium monitoring

**DOI:** 10.1192/pb.bp.113.043752

**Published:** 2014-06

**Authors:** Carol Paton, Thomas R. E. Barnes

**Affiliations:** 1 Prescribing Observatory for Mental Health, Royal College of Psychiatrists’ Centre for Quality Improvement, London; 2 Oxleas NHS Foundation Trust, Dartford; 3 Centre for Mental Health, Charing Cross Campus, Imperial College, London

## Abstract

Audit is an important tool for quality improvement. The collection of data on clinical performance against evidence-based and clinically relevant standards, which are considered by clinicians to be realistic in routine practice, can usefully prompt reflective practice and the implementation of change. Evidence of participation in clinical audit is required to achieve intended learning outcomes for trainees in psychiatry and revalidation for those who are members of the Royal College of Psychiatrists. This article addresses some of the practical steps involved in conducting an audit project, and, to illustrate key points, draws on lessons learnt from a national, audit-based, quality improvement programme of lithium prescribing and monitoring conducted through the Prescribing Observatory for Mental Health.

In 2006, the Chief Medical Officer’s report *Good Doctors, Safer Patients*^1^ called for the reinvigoration of clinical audit to support better patient care and service improvement, and also to be an integral part of the revalidation of clinicians. The Royal College of Psychiatrists’ guidance on revalidation^2^ includes the requirement to participate in clinical audit. For trainees, participation in audit can allow a number of intended learning outcomes^3^ to be achieved. These include gaining an understanding of how clinical governance can be applied in practice, and the development of leadership skills related to monitoring performance and effecting change.

The National Institute for Health and Care Excellence (NICE)^4^ defined clinical audit as a ‘quality improvement process that seeks to improve patient care and outcomes through systematic review of care against explicit criteria and the implementation of change’. This suggests that clinical audit should be seen primarily as a tool for quality improvement. By auditing clinical practice against standards derived from evidence-based guidelines, such as those developed by NICE, defined aspects of the quality of care delivered to patients can be measured and monitored. The Royal College of Psychiatrists’ guidance emphasises that it is not direct involvement in data collection that is important, but rather the review of the audit evidence for the quality of care provided, and taking the lead in implementing strategies to improve quality where these are required. Such reflection on the data by clinicians is perhaps the most potent aspect of clinical audit.

It is important to note that although audit can generate new knowledge, its primary purpose is to compare practice against pre-determined standards. Taking the example of a clinical audit of monitoring of lithium treatment, data would generally be collected directly from clinical records^5^ although questionnaires might be administered for particular purposes, such as gauging patients’ knowledge about the necessary tests or clinicians’ views on responsibility for long-term monitoring. Patients should be receiving usual care. The boundaries between audit, service evaluation and research can be blurred. A brief summary of the differences has been produced by the Royal College of Psychiatrists’ Centre for Quality Improvement^6^ but if there is any doubt about the status of an individual project, advice should be sought from the relevant trust’s audit and research and development departments.

## Choosing an audit topic

Audit is most rewarding when it can drive improvements that have a positive impact on patient care and ultimately on outcomes. As a general rule, if the practice being audited is high volume, high risk or high cost, it is likely to be of importance to the trust as a whole and to patients. Trust priorities for audit are often driven by the requirement to evidence the implementation of NICE guidelines but other legitimate sources include local CQUINS (Commissioning for Quality and Innovation^7^), the requirements of the Care Quality Commission^8^ (CQC), the National Health Service Litigation Authority^9^ (NHSLA), the Mental Health Act 1983 code of practice^10^ or the findings of primary research.

Lithium treatment is a good example of an appropriate focus for audit.^5^ It is a high-volume and high-risk treatment and clear standards can be derived from the NICE guideline for the management of bipolar disorder^11^ and a National Patient Safety alert entitled *Safer Lithium Therapy*.^12^

## Deriving audit standards

The definition of audit includes the use of explicit performance criteria, usually in the form of practice standards. By definition, such standards should be met 100% of the time. It is essential that when standards are defined for an audit they are both evidence based and clinically credible, in that clinicians agree that these standards should always be met in routine clinical practice and it is realistic to do so. Aspirational or controversial standards could undermine the credibility of an audit and limit clinical engagement.

Using lithium treatment again as an example, few clinicians would disagree that checks on renal and thyroid function should be undertaken before lithium is prescribed, that patients should be given information that allows them to use lithium safely (for example, advice on avoiding dehydration and recognising the symptoms of lithium toxicity), and that serum lithium levels should be kept within the therapeutic range. However, many clinicians may disagree with the recommendation from NICE^11^ that serum lithium should be checked in all patients every 3 months. You may therefore decide that 3-monthly monitoring of serum lithium, although not fulfilling criteria for a standard, may still be useful as a clinically relevant ‘treatment target’ measure. This recognises that such frequent monitoring may not be warranted in all patients, but clinicians may still be interested in how their practice in this regard compares with that of their peers.

### Deciding what data to collect

It is important that the audit is practical and feasible in that the clinical practice of interest can be measured reasonably reliably and data collection is not too onerous a task. The data collected must, as a minimum, enable performance against the audit standards to be determined. Contextual data can also be important for analysis of the audit data. For example, with respect to lithium treatment, it is possible that practice will differ between in-patients and out-patients or that monitoring will be more assiduous in elderly people or those with known renal impairment. It is therefore reasonable to collect data related to patient setting, age and renal status. Although the amount and breadth of data collected should be sufficient for understanding the nature and quality of practice, there is also a need to minimise the collection of data that is unlikely to be of use, unlikely to be reliable, particularly time consuming to gather, or likely to be available for only a proportion of the patient sample. If you are unsure, seek advice from the clinical audit department in your trust, rather than spend time collecting information that will be difficult if not impossible to interpret in the context of the audit.

### Developing a data-collection tool

Effective data-collection tools are time consuming and difficult to design. The question for each data field needs to be as clear and unambiguous as possible so that there is no doubt about what is being measured. If there is any ambiguity, it is probable that data will be collected in an unstandardised way and will therefore be uninterpretable. For example, if you were interested in the monitoring of renal function in patients treated with lithium, it would be best to ask explicitly whether a measure of estimated glomerular filtration rate or creatinine clearance were documented in the clinical records. A vague, poorly worded question could lead to a positive answer on the assessment of renal function even though the assessment had been limited to investigation of electrolytes and urea, or an entry in the clinical records stating that investigations had been requested but without any documented results.

## Selecting an audit sample

The audit standards should guide the selection of the sample. For example, if you were interested in whether screening tests were conducted before lithium treatment was initiated, it might be most appropriate to identify a sample from acute in-patient wards. If the focus were monitoring of established treatment, a community sample might be more suitable. Your trust’s informatics department may be able to give you a list of all patients with a given diagnosis but are unlikely to be able to identify only those prescribed lithium. You may be able to obtain a list of patients prescribed lithium through a local lithium clinic, pharmacy dispensing records or a manual search of in-patient prescription charts. Each method has limitations and some methods will require help from other members of staff/departments. Make sure you plan this in good time.

## Organising data collection

Estimate the time it will take to collect the data you require and test this by piloting the data-collection tool on a small sample of cases. This will serve the dual purpose of determining whether the questions are clear and unambiguous, and whether the data you require can be found in a timely manner. If it seems unrealistic for you to complete the planned data collection on your own, you may decide to restrict the focus of the audit or enlist help from someone with the appropriate experience, skills and knowledge.

The source(s) of data should be decided in advance, determined by the audit standards chosen. For example, in a patient recently initiated on lithium treatment, searching the clinical record for an entry that stated the patient had been given information about the therapy would be an audit of documentation of clinical practice. If you then asked the clinical team whether the patient had been given a copy of the National Patient Safety Agency patient lithium pack or local alternative, this would be a direct audit of clinical practice.

## Analysing the data and interpreting the findings

For the majority of audits it is likely that the data collected will need to be entered onto an electronic spreadsheet to facilitate the generation of charts and figures and, if appropriate, to allow the use of a statistical package. Data entry is time consuming and error prone, and the accuracy of the entered data will need to be checked.

It is advisable to follow a formal analysis plan, which has been determined by the audit questions. It is possible to spend a great deal of time conducting analyses that are not appropriate, and if you need help or advice, this may be requested from your trust’s clinical audit, research and development or informatics departments.

With respect to stand-alone, local audits, it can be difficult to interpret the findings, particularly if the sample size is small. For example, if you were to find that only 80% of patients had a check of renal function before starting lithium treatment you might be uncertain about whether this represented good, bad or indifferent practice. Although clearly short of adherence with the audit standard, such a proportion would be put in context by comparison with equivalent data from other services and an understanding of the clinical variables associated with performance on the standard. This is possible with participation in a national audit of lithium monitoring, such as that organised by the Prescribing Observatory for Mental Health^5,13^ (POMH-UK), where a standard data-collection tool is used across the country and the data are analysed to provide benchmarking across trusts and between clinical teams within trusts. Such national audits, conducted for the purpose of quality improvement, generate data intended for internal use within a clinical team or trust to prompt reflective practice and local learning. The data may not be robust enough to be suitable for ranking or judgement^14^ and it is important that this is understood by those who wish to use or act on the audit findings.

## Disseminating an audit report

It is important to consider who needs to see the audit findings and why. The data will be more meaningful to clinical teams if they are presented in a clear, succinct, comprehensible and accessible manner. If the main aim of the audit is to assess performance against a few key standards, try to ensure that such performance is clearly illustrated and not buried among other less relevant findings. Feedback to individual clinical teams should be prompt so that it represents current practice, and is not at risk of being dismissed as irrelevant because of subsequent changes to the service. For example, the results of a local audit of lithium monitoring on a small patient sample, conducted several months ago, may be seen as only of historical interest if there have been subsequent changes such as the introduction of a local lithium database, a change in case-load for the clinical team or the appointment of a new consultant. Prioritising early dissemination of the audit findings to the participating teams is likely to have more impact on clinical practice than taking a more formal and inevitably slower route through the trust’s governance structures.

## Changing clinical practice

Changing clinical practice can be a difficult and slow process. Experience from audit-based quality improvement programmes conducted by POMH-UK suggests that it can take up to 3 years to see change at a national level, and that the magnitude of that change is generally modest.^15,16^ However, within the context of the POMH-UK programmes some individual services have implemented change more quickly and sustained the improvements made. Enabling factors include the active engagement of clinical teams in the audit process, as mentioned earlier, having a local champion who supports and promotes the audit, effective trust systems and infrastructure, and an organisational culture of audit and quality improvement. Incentives for the participation of psychiatrists include a desire to provide best care, the use of audit evidence to support revalidation and, for trainees, the achievement of intended learning outcomes. Barriers to change include concern about being exposed to external judgement, the audit findings not being disseminated to participating clinical teams, limited support or capacity to develop action plans and effect change, and staff changes or service reorganisations that almost inevitably result in a loss of continuity and momentum. The potential for change will also be limited if a short-term view is taken and the audit cycle is not completed. To maximise the clinical impact of any audit it is important that the cycle is followed: set standards, measure, reflect, implement change, review standards, measure, reflect, etc. It may take several cycles, testing and refining change interventions, to make and embed improvements in clinical practice.
